# Supplementary material for: C-STABILITY an innovative modeling framework to leverage the continuous representation of organic matter
Source: Nat Commun. 2021 Feb 5;12:810. doi: 10.1038/s41467-021-21079-6 (PMC7864906; doi:10.1038/s41467-021-21079-6)
Supplement: Supplementary file 1 — Supplementary Information [file 41467_2021_21079_MOESM1_ESM.pdf]

C-STABILITY an innovative modeling framework to leverage the continuous representation of organic matter (supplementary information)

Sainte-Marie Julien *[1,2,3], Barrandon Matthieu[4], Saint-André Laurent[2,3], Gelhaye Eric[3,5], Martin Francis[3,5], and Derrien Delphine †[2,3]

[1]Université de Lorraine, AgroParisTech, INRAE, SILVA, F-54000 Nancy, France
[2]INRAE, BEF, F-54000 Nancy, France
[3]Laboratory of Excellence ARBRE, F-54000 Nancy, France
[4]Université de Lorraine, CNRS, IECL, F-54000 Nancy, France
[5]Université de Lorraine, INRAE, IAM, F-54000 Nancy, France

*julien.sainte-marie@agroparistech.fr
†delphine.derrien@inrae.fr

Supplementary note 1

Table 1: Studies utilized to design the scenarios.

Scenarios 1 & 2	Concurrent changes in substrate chemistry and enzymes occurence/ enzyme efficiency in laboratory litter decomposition	[1,2,3,4,5]
Scenario 3	Classification of decomposers into functional communities	[6,7,8,9]
	Successions of decomposer communities during in situ decomposition of plant litter	[10,11,12,13]
Scenario 4	Chemical characterisation of organic biomolecules present in soil organic matter	[14,15,16]

Supplementary note 2

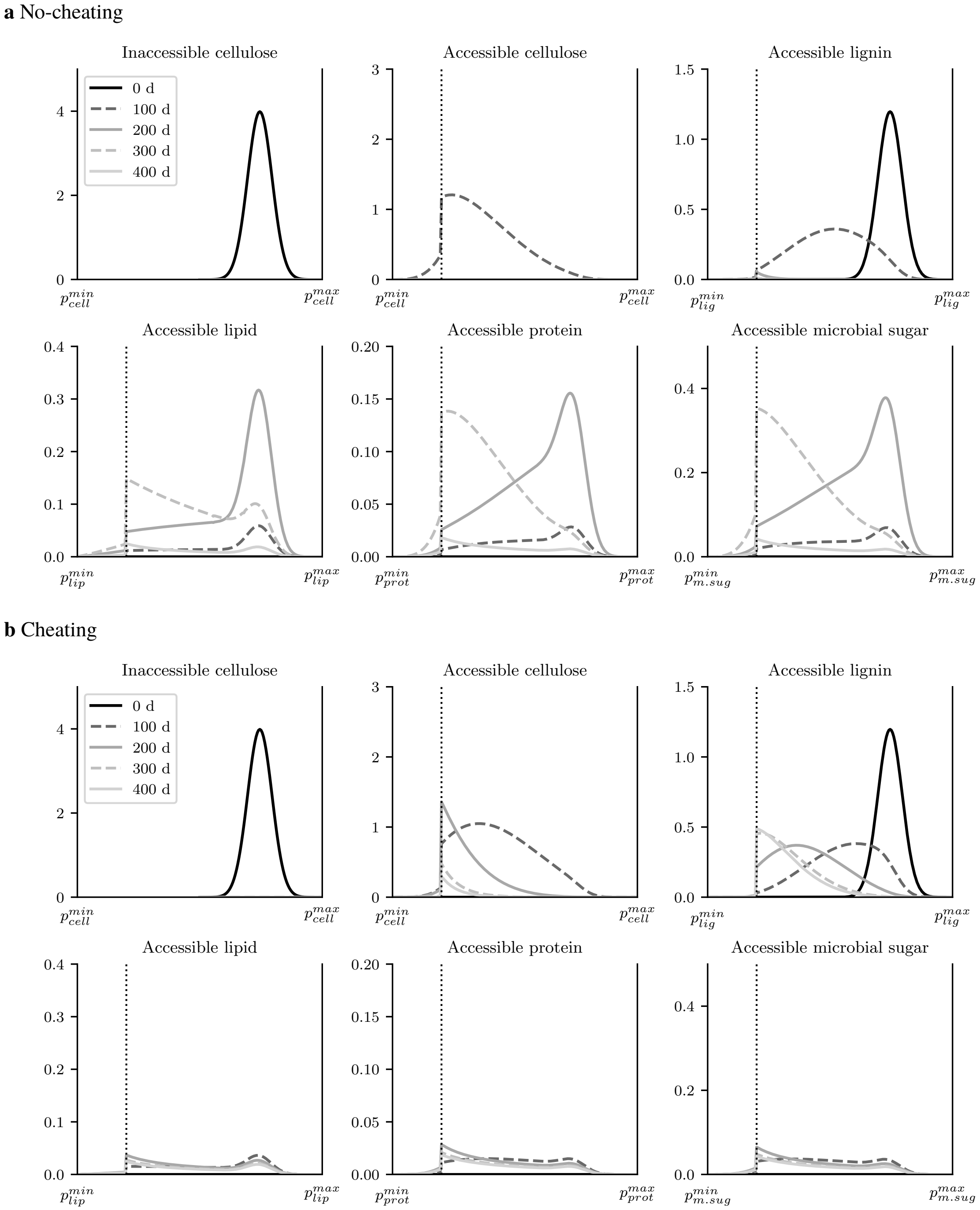

Fig. 1: **Temporal variations in polymerization distribution for each substrate pool in scenario 3**. A comparison between **a** no-cheating and **b** cheating microbial behaviour.

Supplementary note 3

Introduction We give here a proof of steady state equations for scenario 4. We recall the general equations of the system.

$$\begin{aligned}
\frac{\partial \chi_*^{ac}}{\partial t}(p,t) &= \tau_{tr,*}^{ac}\chi_*^{in}(p,t) - \tau_{tr,*}^{in}\chi_*^{ac}(p,t) \\
&\quad -\tau_{enz}^{0}\sum_{\text{mic}} C_{mic}(t)\chi_*^{ac}(p,t) \\
&\quad +\tau_{enz}^{0}\sum_{\text{mic}} C_{mic}(t)(\alpha_{enz}+1)(p-p_*^{min})^{\alpha_{enz}}\int_p^{p_*^{max}} \frac{\chi_*^{ac}(p',t)}{(p'-p_*^{min})^{\alpha_{enz}+1}}dp' \\
&\quad +\sum_{\text{mic}} C_{mic}(t)\left(m_{mic}^{0}s_{mic,*}(p) - \mathbb{1}_{\mathcal{D}_u}(p)u_{mic,*}^{0}\chi_*^{ac}(p,t)\right) \\
&\quad +i_*^{ac}(p,t), && \text{(S1)}\\
\frac{\partial \chi_*^{in}}{\partial t}(p,t) &= \tau_{tr,*}^{in}\chi_*^{ac}(p,t) - \tau_{tr,*}^{ac}\chi_*^{in}(p,t) + i_*^{in}(p,t). && \text{(S2)}
\end{aligned}$$

where $\mathbb{1}_{\mathcal{D}_u}(p)$ equals 1 if $p \in \mathcal{D}_u$ and 0 otherwise. The dynamics of microbial C_{mic} is obtained by,

$$\frac{dC_{mic}}{dt}(t) = -m_{mic}^{0}C_{mic}(t) + \sum_{*} u_{mic,*}^{0}e_{mic,*}^{0}C_{mic}(t)\int_{\mathcal{D}_u}\chi_*^{ac}(p,t)dp. \quad \text{(S3)}$$

Specific assumptions Due to the specific design of the scenario 4, several terms of the equations can be simplified. First, we consider a single microbial community and all microbial parameters (u^0, e^0 and m^0) are considered identical for all biochemical classes. Second, we consider that all the substrate is accessible i.e. $\chi_*^{in} = 0$ and $\tau_{tr,*}^{ac} = \tau_{tr,*}^{in} = \iota_*^{in} = 0$. Then all distinction between accessible and inaccessible substrate were removed in the following. Third, we consider that input terms $\iota_*^{ac}(p,t) = \iota_*^{ac}(p)$ are constant over time. To improve readability of the proof, we note without ambiguity $\alpha_{enz} = \alpha_*$ and $\tau_{enz}^{0} = \tau_*^{0}$ in the following. Then, for all biochemical classes $*$, we have for all $p \in [p_*^{min}, p_*^{max}]$,

$$\begin{aligned}
\frac{\partial \chi_*}{\partial t}(p,t) &= -\tau_*^{0}C_{mic}(t)\chi_*(p,t) \\
&\quad +\tau_*^{0}C_{mic}(t)(\alpha_*+1)(p-p_*^{min})^{\alpha_*}\int_p^{p_*^{max}} \frac{\chi_*(p',t)}{(p'-p_*^{min})^{\alpha_*+1}}dp' \\
&\quad +C_{mic}(t)\left(m^{0}s_*(p) - \mathbb{1}_{\mathcal{D}_u}(p)u^{0}\chi_*(p,t)\right) \\
&\quad +i_*(p), && \text{(S4)}\\
\frac{dC_{mic}}{dt}(t) &= -m^{0}C_{mic}(t) + u^{0}e^{0}C_{mic}(t)\sum_{*}\int_{\mathcal{D}_u}\chi_*(p,t)dp. && \text{(S5)}
\end{aligned}$$

Integral operators We introduce $p_*^{u} \in [p_*^{min}, p_*^{max}]$ such that $\mathcal{D}_u = [p_*^{min}, p_*^{u}]$ and we define the operators A_* for a function $f :]a, p_*^{max}] \to \mathbb{R}$ with $p_*^{min} \leq a$,

$$A_*f(p) = (\alpha_*+1)(p-p_*^{min})^{\alpha_*}\int_p^{p_*^{max}} \frac{f(p')}{(p'-p_*^{min})^{\alpha_*+1}}dp', \quad \text{(S6)}$$

and A_*^{u} for a function $f :]a', p_*^{u}] \to \mathbb{R}$ with $p_*^{min} \leq a'$,

$$A_*^{u}f(p) = (\alpha_*+1)(p-p_*^{min})^{\alpha_*}\int_p^{p_*^{u}} \frac{f(p')}{(p'-p_*^{min})^{\alpha_*+1}}dp'. \quad \text{(S7)}$$

Steady-state equations If we set $\frac{\partial \chi_*}{\partial t}(p,t) = 0$ and $\frac{dC_{mic}}{dt}(t) = 0$, we obtain the following equations to solve to obtain $\chi_*(p)$ and C_{mic} steady state expressions for all $p \in [p_*^{min}, p_*^{max}]$,

$$\frac{\iota_*(p)}{C_{mic}} = \tau_*^0 \left(\chi_*(p) - A_*\chi_*(p)\right) + \mathbb{1}_{\mathcal{D}_u}(p)u^0\chi_*(p) - m^0 s_*(p), \tag{S8}$$

$$\frac{m^0}{e^0} = u^0 \sum_* \int_{p_*^{min}}^{p_*^u} \chi_*(p)dp. \tag{S9}$$

Determination of C_{mic} We consider the integration over $p \in [p_*^{min}, p_*^{max}]$ of equation S8 which gives,

$$\frac{I_*}{C_{mic}} = \tau_*^0 \int_{p_*^{min}}^{p_*^{max}} \left(\chi_*(p) - A_*\chi_*(p)\right) dp + u^0 \int_{p_*^{min}}^{p_*^u} \chi_*(p)dp - m^0 S_*.$$

We can verify that $\int_{p_*^{min}}^{p_*^{max}} \left(\chi_*(p) - A_*\chi_*(p)\right) dp = 0$ and if we sum over the biochemical classes $*$,

$$\frac{I}{C_{mic}} = u^0 \sum_* \int_{p_*^{min}}^{p_*^u} \chi_*(p)dp - m^0.$$

According to equation S9,

$$C_{mic} = \frac{Ie^0}{m^0(1-e^0)}. \tag{S10}$$

Determination of χ_* We define $\theta_*(p) = m^0 s_*(p) + \frac{\iota_*(p)}{C_{mic}}$ and then, we can reformulate equation S8,

$$A_*\chi_*(p) - \chi_*(p) = -\frac{\theta_*(p)}{\tau_*^0}, \qquad \text{for all } p \in]p_*^u, p_*^{max}] \tag{S11}$$

$$A_*\chi_*(p) - \frac{\tau_*^0 + u^0}{\tau_*^0}\chi_*(p) = -\frac{\theta_*(p)}{\tau_*^0}, \qquad \text{for all } p \in [p_*^{min}, p_*^u] \tag{S12}$$

To solve these equations, we study integral operators A_* and A_*^u and due to their formalism analogies, we only consider A. We verify that A_*f satisfy the equation,

$$(A_*f)'(p) = \frac{\alpha_*}{p - p_*^{min}} A_*f(p) - \frac{\alpha_* + 1}{p - p_*^{min}} f(p),$$

Let be $\lambda \in \mathbb{R}$ and $F :]a, p_*^{max}] \to \mathbb{R}$, we consider the equation to solve,

$$A_*f - \lambda f = F,$$

and by assuming that F can be differentiated, we obtain,

$$\frac{\alpha_*}{p - p_*^{min}} A_*f(p) - \frac{\alpha_* + 1}{p - p_*^{min}} f(p) - \lambda f'(p) = F'(p).$$

As $A_*f = \lambda f + F$, we obtain,

$$\frac{\lambda\alpha_* - (\alpha_* + 1)}{p - p_*^{min}} f(p) - \lambda f'(p) = F'(p) + \frac{\alpha_*}{p - p_*^{min}} F(p),$$

By defining $\delta_* = \frac{\alpha_* + 1 - \lambda\alpha_*}{\lambda}$, we obtain,

$$f'(p) + \frac{\delta}{p - p_*^{min}} f(p) = -\frac{1}{\lambda}F'(p) + \frac{\alpha_*}{\lambda(p - p_*^{min})} F(p). \tag{S13}$$

The general solution of the ordinary differential equation S13 is,

$$f(p)=\frac{c}{(p-p_*^{min})^{\delta_*}}-\frac{1}{\lambda}F(p)-\frac{\alpha_*+1}{\lambda^2(p-p_*^{min})^{\delta_*}}\int_p^{p_*^{max}}(p'-p_*^{min})^{\delta_*-1}F(p')dp'.$$

We remark that $c=0$ because $A_*f(p_*^{max})=0$ then,

$$f(p)=-\frac{1}{\lambda}F(p)-\frac{\alpha_*+1}{\lambda_*^2(p-p_*^{min})^{\delta_*}}\int_p^{p_*^{max}}(p'-p^{min})^{\delta_*-1}F(p')dp'. \quad \text{(S14)}$$

In addition, we can verify that this result remains true even if F cannot be differentiated. We easily obtain the result of equation S11 from equation S14 by setting $\lambda=1$, which gives $\delta_*=1$, and $F(p)=-\frac{\theta_*(p)}{\tau_*^0}$. Then, for all $p\in]p_*^u,p_*^{max}]$,

$$\chi_*(p)=\frac{\theta_*(p)}{\tau_*^0}+\frac{\alpha_*+1}{(p-p_*^{min})}\int_p^{p_*^{max}}\frac{\theta_*(p')}{\tau_*^0}dp'. \quad \text{(S15)}$$

From now, we consider the equation S12. In particular, we have,

$$A_*\chi_*(p)=A_*^u\chi_*(p)+(\alpha_*+1)(p-p_*^{min})^{\alpha_*}\int_{p_*^u}^{p_*^{max}}\frac{\chi_*(p')}{(p'-p_*^{min})^{\alpha_*+1}}dp',$$

and by using the result obtained in equation S15,

$$A_*\chi_*(p)=A_*^u\chi_*(p)+\frac{(\alpha_*+1)(p-p_*^{min})^{\alpha_*}}{(p_*^u-p_*^{min})^{\alpha_*+1}}\int_{p_*^u}^{p_*^{max}}\frac{\theta_*(p')}{\tau_*^0}dp',$$

then, if we define $\gamma=\frac{\alpha_*+1}{(p_*^u-p_*^{min})^{\alpha_*+1}}\int_{p_*^u}^{p_*^{max}}\frac{\theta_*(p')}{\tau_*^0}dp'$, equation S12 becomes for all $p\in[p_*^{min},p_*^u]$,

$$A_*^u\chi_*(p)-\frac{\tau_*^0+u^0}{\tau_*^0}\chi_*(p)=-\frac{\theta_*(p)}{\tau_*^0}-(p-p_*^{min})^{\alpha_*}\gamma. \quad \text{(S16)}$$

To solve this equation, we use the same process as previously by considering a solution of $A_*^uf-\lambda f=F$ by setting $\lambda=\frac{\tau_*^0+u^0}{\tau_*^0}$, which gives $\delta_*=\frac{\tau_*^0-u^0\alpha_*}{\tau_*^0+u^0}=\beta_*$, and $F(p)=-\frac{\theta_*(p)}{\tau_*^0}-\gamma(p-p_*^{min})^{\alpha_*}$. We obtain for all $p\in[p_*^{min},p_*^u]$,

$$\chi_*(p)=-\frac{\tau_*^0}{\tau_*^0+u^0}F(p)-\frac{\tau_*^{0\,2}(\alpha_*+1)}{(\tau_*^0+u^0)^2(p-p_*^{min})^{\beta_*}}\int_p^{p_*^u}(p'-p_*^{min})^{\beta_*-1}F(p')dp'.$$

By replacing explicitly F by its formulation, we obtain,

$$\begin{aligned}\chi_*(p) &= \frac{\theta_*(p)}{\tau_*^0+u^0}+\frac{\tau_*^0(\alpha_*+1)}{(\tau_*^0+u^0)^2(p-p_*^{min})^{\beta_*}}\int_p^{p_*^u}(p'-p_*^{min})^{\beta_*-1}\theta_*(p')dp'\\ &\quad+\frac{\gamma\tau_*^0}{\tau_*^0+u^0}(p-p_*^{min})^{\alpha_*}+\frac{\gamma\tau_*^{0\,2}(\alpha_*+1)}{(\tau_*^0+u^0)^2(p-p_*^{min})^{\beta_*}}\int_p^{p_*^u}(p'-p_*^{min})^{\alpha_*+\beta_*-1}dp',\\ \chi_*(p) &= \frac{\theta_*(p)}{\tau_*^0+u^0}+\frac{\tau_*^0(\alpha_*+1)}{(\tau_*^0+u^0)^2(p-p_*^{min})^{\beta_*}}\int_p^{p_*^u}(p'-p_*^{min})^{\beta_*-1}\theta_*(p')dp'\\ &\quad+\frac{\gamma\tau_*^0}{\tau_*^0+u^0}(p-p_*^{min})^{\alpha_*}+\frac{\gamma\tau_*^{0\,2}(\alpha_*+1)\left((p_*^u-p_*^{min})^{\alpha_*+\beta_*}-(p-p_*^{min})^{\alpha_*+\beta_*}\right)}{(\tau_*^0+u^0)^2(\alpha_*+\beta_*)(p-p_*^{min})^{\beta_*}}.\end{aligned}$$

We remark that $\alpha_* + \beta_* = \frac{(\alpha_*+1)\tau_*^0}{\tau_*^0+u^0}$. Then,

$$\begin{aligned}
\chi_*(p) &= \frac{\theta_*(p)}{\tau_*^0+u^0} + \frac{\tau_*^0(\alpha_*+1)}{(\tau_*^0+u^0)^2(p-p_*^{min})^{\beta_*}} \int_p^{p_*^u} (p'-p_*^{min})^{\beta_*-1}\theta_*(p')dp' \\
&\quad + \frac{\gamma\tau_*^0}{\tau_*^0+u^0}(p-p_*^{min})^{\alpha_*} + \frac{\gamma\tau_*^0\left((p_*^u-p_*^{min})^{\alpha_*+\beta_*}-(p-p_*^{min})^{\alpha_*+\beta_*}\right)}{(\tau_*^0+u^0)(p-p_*^{min})^{\beta_*}}, \\
\chi_*(p) &= \frac{\theta_*(p)}{\tau_*^0+u^0} + \frac{\tau_*^0(\alpha_*+1)}{(\tau_*^0+u^0)^2(p-p_*^{min})^{\beta_*}} \int_p^{p_*^u} (p'-p_*^{min})^{\beta_*-1}\theta_*(p')dp' \\
&\quad + \frac{\tau_*^0}{\tau_*^0+u^0}(p-p_*^{min})^{\alpha_*}\left(1+\frac{(p_*^u-p_*^{min})^{\alpha_*+\beta_*}-(p-p_*^{min})^{\alpha_*+\beta_*}}{(p-p_*^{min})^{\alpha_*+\beta_*}}\right)\gamma, \\
\chi_*(p) &= \frac{\theta_*(p)}{\tau_*^0+u^0} + \frac{\tau_*^0(\alpha_*+1)}{(\tau_*^0+u^0)^2(p-p_*^{min})^{\beta_*}} \int_p^{p_*^u} (p'-p_*^{min})^{\beta_*-1}\theta_*(p')dp' \\
&\quad + \frac{\tau_*^0}{\tau_*^0+u^0}\frac{(p_*^u-p_*^{min})^{\alpha_*+\beta_*}}{(p-p_*^{min})^{\beta_*}}\gamma.
\end{aligned}$$

Finally, by replacing explicitly γ by its formulation, we obtain for all $p \in [p_*^{min}, p_*^u]$,

$$\begin{aligned}
\chi_*(p) = &\frac{\theta_*(p)}{\tau_*^0+u^0} + \frac{\tau_*^0(\alpha_*+1)}{(\tau_*^0+u^0)(p-p_*^{min})^{\beta_*}} \int_p^{p_*^u} (p'-p_*^{min})^{\beta_*-1}\frac{\theta_*(p')}{\tau_*^0+u^0}dp' \\
&+(\alpha_*+1)\frac{(p_*^u-p_*^{min})^{\beta_*-1}}{(p-p_*^{min})^{\beta_*}}\int_{p_*^u}^{p_*^{max}}\frac{\theta_*(p')}{\tau_*^0+u^0}dp'.
\end{aligned} \tag{S17}$$

Carbon stocks Based on equations S15 and S17, we can compute the amounts of C per pool and domain. The amount of carbon inaccessible to carbon uptake $C_{*,\bar{\mathcal{D}}_u}$ is,

$$\begin{aligned}
C_{*,\bar{\mathcal{D}}_u} =& \int_{p_*^u}^{p_*^{max}} \chi_*(p)dp \\
=& \frac{m^0}{\tau_*^0}\int_{p_*^u}^{p_*^{max}}\left(1+(\alpha_*+1)ln\left(\frac{p-p_*^{min}}{p_*^u-p_*^{min}}\right)\right)s_*(p)dp \\
&+\frac{m^0(1-e^0)}{\tau_*^0e^0I}\int_{p_*^u}^{p_*^{max}}\left(1+(\alpha_*+1)ln\left(\frac{p-p_*^{min}}{p_*^u-p_*^{min}}\right)\right)i_*(p)dp,
\end{aligned} \tag{S18}$$

and the amount of carbon accessible to carbon uptake $C_{*,\mathcal{D}_u}$ is,

$$\begin{aligned}
C_{*,\mathcal{D}_u} =& \int_{p_*^{min}}^{p_*^u} \chi_*(p)dp \\
=& \frac{m^0}{u^0}\int_{p_*^{min}}^{p_*^{max}} s_*(p)dp + \frac{m^0(1-e^0)}{u^0e^0I}\int_{p_*^{min}}^{p_*^{max}} i_*(p)dp.
\end{aligned} \tag{S19}$$

Supplementary References

[1] Kaffenberger, J. T. & Schilling, J. S. Comparing lignocellulose physiochemistry after decomposition by brown rot fungi with distinct evolutionary origins. *Environmental Microbiology* **17**, 4885–4897 (2015).

[2] Kuuskeri, J. *et al.* Time-scale dynamics of proteome and transcriptome of the white-rot fungus phlebia radiata: growth on spruce wood and decay effect on lignocellulose. *Biotechnology for biofuels* **9**, 1–22 (2016).

[3] Lashermes, G., Gainvors-Claisse, A., Recous, S. & Bertrand, I. Enzymatic strategies and carbon use efficiency of a litter-decomposing fungus grown on maize leaves, stems, and roots. *Frontiers in Microbiology* **7** (2016).

[4] Skyba, O., Douglas, C. J. & Mansfield, S. D. Syringyl-rich lignin renders poplars more resistant to degradation by wood decay fungi. *Applied and Environmental Microbiology* **79**, 2560–2571 (2013).

[5] Zhang, J. *et al.* Localizing gene regulation reveals a staggered wood decay mechanism for the brown rot fungus postia placenta. *Proceedings of the National Academy of Sciences of the United States of America* **113**, 10968–10973 (2016).

[6] Kohler, A. *et al.* Convergent losses of decay mechanisms and rapid turnover of symbiosis genes in mycorrhizal mutualists. *Nature Genetics* **47**, 410–U176 (2015).

[7] Miyauchi, S. *et al.* Large-scale genome sequencing of mycorrhizal fungi provides insights into the early evolution of symbiotic traits. *Nature Communications* **11**, 1–17 (2020).

[8] Nagy, L. G. *et al.* Genetic bases of fungal white rot wood decay predicted by phylogenomic analysis of correlated gene-phenotype evolution. *Molecular Biology and Evolution* **34**, 35–44 (2016).

[9] Talbot, J. M., Martin, F., Kohler, A., Henrissat, B. & Peay, K. G. Functional guild classification predicts the enzymatic role of fungi in litter and soil biogeochemistry. *Soil Biology and Biochemistry* **88**, 441–456 (2015).

[10] Rajala, T., Peltoniemi, M., Hantula, J., Mäkipää, R. & Pennanen, T. Rna reveals a succession of active fungi during the decay of norway spruce logs. *Fungal Ecology* **4**, 437–448 (2011).

[11] Schneider, T. *et al.* Who is who in litter decomposition? metaproteomics reveals major microbial players and their biogeochemical functions. *Isme Journal* **6**, 1749–1762 (2012).

[12] Snajdr, J. *et al.* Transformation of quercus petraea litter: successive changes in litter chemistry are reflected in differential enzyme activity and changes in the microbial community composition. *Fems Microbiology Ecology* **75**, 291–303 (2011).

[13] Tláskal, V., Vorísková, J. & Baldrian, P. Bacterial succession on decomposing leaf litter exhibits a specific occurrence pattern of cellulolytic taxa and potential decomposers of fungal mycelia. *Fems Microbiology Ecology* **92** (2016).

[14] Kallenbach, C. M., Frey, S. D. & Grandy, A. S. Direct evidence for microbial-derived soil organic matter formation and its ecophysiological controls. *Nature Communications* **7** (2016).

[15] Kohl, L. *et al.* The origin of soil organic matter controls its composition and bioreactivity across a mesic boreal forest latitudinal gradient. *Global Change Biology* **24**, E458–E473 (2018).

[16] Vancampenhout, K. *et al.* Differences in chemical composition of soil organic matter in natural ecosystems from different climatic regions–a pyrolysis–gc/ms study. *Soil Biology and Biochemistry* **41**, 568–579 (2009).
