# Supplementary material for: C-STABILITY an innovative modeling framework to leverage the continuous representation of organic matter
Source: Nat Commun. 2021 Feb 5;12:810. doi: 10.1038/s41467-021-21079-6 (PMC7864906; doi:10.1038/s41467-021-21079-6)
Supplement: Supplementary file 4 — Description of additional supplementary files [file 41467_2021_21079_MOESM4_ESM.doc]

Description of additional supplementary information

**Title: Supplementary movie**

Description: A commented video entitled “C-STABILITY\_lignocellulose.mp4” illustrates scenario 2 lignocellulose degradation.
